# Effects of online continuing medical education on perspectives of shared decision-making among Chinese endocrinologists

**DOI:** 10.1186/s12909-023-04838-5

**Published:** 2023-11-17

**Authors:** Hongbo Yang, Shi Chen, Nan Zhao, Xiang Zhou, Lijia Cui, Weibo Xia, Yuxiu Li, Huijuan Zhu

**Affiliations:** 1 Key Laboratory of Endocrinology of National Health Commission, Department of Endocrinology, Peking Union Medical College Hospital, Chinese Academy of Medical Science and Peking Union Medical College, 100730 Beijing, China; 2 Medical Research Center, State Key Laboratory of Complex Severe and Rare Diseases, Peking Union Medical College Hospital, Chinese Academy of Medical Sciences and Peking Union Medical College, Beijing, China

**Keywords:** Shared decision-making, Perspective, Endocrinology, National survey, Continued medical education

## Abstract

**Background:**

Shared decision-making (SDM) may influence the clinical outcomes of patients with endocrine disorders. There are few studies describing perspectives towards SDM among endocrinologists in China.

**Methods:**

In the first stage, we conducted a national survey using an online questionnaire about SDM among endocrinologists in China. The national survey focused on attitude and propensity, potential barriers, and the effectiveness of SDM implementation strategies. In the second stage, survey participants were further recruited to participate in a prospective cohort study in the online continuing medical education (CME) program of Peking Union Medical College Hospital in Beijing. The Shared Decision-Making Questionnaire (SDM-Q-Doc) was employed to assess the effects of online CME on physicians’ perspectives during the process of SDM, which was conducted before and after the CME course was provided.

**Results:**

In the national survey, 280 endocrinologists (75.7% female, mean age 38.0 ± 4.5 years, 62.5% with a duration of practice of more than ten years) completed the questionnaire. Participants had a generally positive attitude towards SDM in clinical practice. The main perceived barriers included time consumption, information inequality between doctors and patients, and a lack of technical support and training for SDM. The main uncertainties of implementation steps included inviting patients to participate in SDM (16.3%), assisting in decision-making (15.3%), facilitating deliberation and decision-making (13.7%), and providing information on benefits and risks (12.6%). Of the physicians who participated in the national survey, 84 registered for the eight-day online CME course. The SDM-Q-Doc score increased from 87.3 ± 18.2 at baseline to 93.0 ± 9.3 at the end of the 8-day online CME training (p = 0.003, paired t test). The participants’ age, sex, education level, practice duration, the annual number of patients with rare endocrine diseases, and the annual number of patients requiring MDT or CME were not significantly related to increased SDM-Q-Doc scores after online CME (all p > 0.05).

**Conclusions:**

Chinese endocrinologists had a generally positive attitude towards SDM in clinical practice. There were also several uncertainties in the implementation steps of SDM. Regardless of a physician’s educational background or prior professional experience, CME may help to improve their perspectives regarding SDM.

## Background

Shared decision-making (SDM) is an interactive mode in which physicians and patients exchange information, personal values, and preferences equally and participate together to reach a consensus about clinical management [1]. Shared decision-making could decrease decisional conflict, promote the congruence of goals and options, and encourage patient involvement [2]. SDM is promoted in many health care systems and medical specialties since it effectively improves clinical outcomes [3, 4].

The management of endocrine disorders is complicated by long-term courses, indefinite prognoses, and feelings of isolation among patients [5]. Integrating SDM into routine practice is imperative to ensure that all risks and benefits of treatment are fully discussed and weighed with the patient’s expectations and goals in mind. Recently, some studies have investigated the contribution of SDM to improving medical outcomes during the diagnosis and treatment of patients with endocrine disorders [6]. In China, studies about the implementation and evaluation of SDM in clinical endocrinology have yet to be conducted.

There are various barriers to the implementation of SDM in clinical practice, including clinician attitudes, professional environment, time constraints, and a lack of supporting resources [7, 8]. Training for physicians and patients is essential in improving SDM outcomes [9]. The training of doctors includes professional training and SDM theory and practice training. The former improves the self-confidence of doctors in clinical practice through professional knowledge training, and the latter improves their competence in SDM through technical training [10]. Whether continuing medical education focusing on the most recent developments in the clinical management of endocrine and metabolic disorders could improve competence in SDM among specialists with years of clinical experience needs more investigation.

In the present study, we conducted a national survey to investigate Chinese endocrinologists’ perspectives regarding SDM in managing endocrine diseases. We further evaluated the effect of continuing education on attitudes regarding SDM in a prospective cohort study.

## Methods

### Study design

In the first stage, we conducted a national survey using an online questionnaire about SDM among endocrinologists in June 2022. The study was carried out in accordance with the Checklist for Reporting Findings of Internet E-surveys (CHERRIES) requirements [11]. Closed questions were employed to allow the participants to choose from a number of alternative answers. No questions should be skipped or answered only in part.

**Fig. 1:**
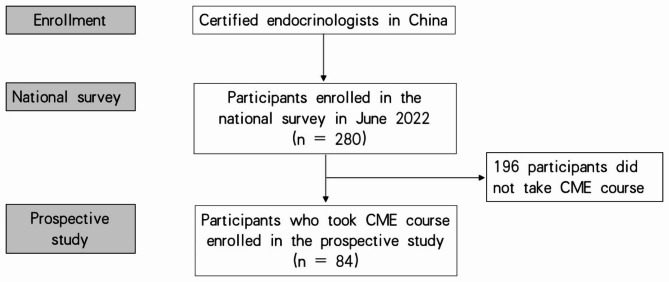
Study Design and Flow Diagram

In the second stage in July 2022, survey participants were further recruited to participate in a prospective cohort study involving the online CME program of Peking Union Medical College Hospital in Beijing, China (Fig. 1).

### National survey

The inclusion criterion was registered specialists of the Endocrinology branch of the Chinese Medical Association. The exclusion criteria were as follows: (1) specialists engaged in other professions and (2) those who were unable to complete the questionnaire survey using a mobile phone.

The national survey was conducted using the mobile WeChat terminal and the internet survey platform ‘Questionnaire Star’ (http://www.wjx.cn) [12]. The snowball sampling method was used to invite endocrinologists to participate in the national survey [13]. The questionnaire could only be answered through the WeChat platform, and each participant had a unique WeChat account and could only complete the questionnaire once. A reminder was sent to complete the questionnaire via WeChat during the national survey since all responders answered all the questions with unique WeChat accounts.

There was a total of 25 questions in the survey (Table 1). Data were automatically collected through the website. Before submitting their answers, participants could review them. The main contents of the survey included the following:

**Table 1:** The 25 questions on the national survey

Item	Question
1	What is your age?
2	What is your sex?
3	What is your highest degree?
4	How long have you been engaged in endocrine clinical work?
5	What is your professional title?
6	Are you currently working at a teaching hospital?
7	What is the approximate number of cases of rare endocrine diseases that you treat every year?
8	How many times do you participate in the multidisciplinary diagnosis and treatment of patients with rare endocrine diseases every year?
9	How many times do you receive online/offline academic training on the diagnosis and treatment of rare endocrine diseases each year?
10	Do you invite patients to participate in medical decision-making during the diagnosis and treatment of rare endocrine diseases?
11	Do you provide patients with detailed information on the available treatment options?
12	Would you say to a patient, ‘Different people have different considerations, and among the possible benefits and risks of these plans, which outcomes do you expect to achieve? Which outcomes are your concerns?’
13	After clarifying a patient’s preferences for treatment, would you help them weigh the pros and cons of possible options?
14	During the diagnosis and treatment process, would you say to a patient, ‘Let’s discuss what needs to be done next?’
15	Do you use doctor‒patient collaborative decision-making network resources in your daily work?
16	What do you think are the obstacles to making appropriate joint decisions with patients?
For question 17 to question 25, a scene of an adult patient with Turner syndrome is described. The patient is unwilling to continue treatment due to concerns about the impact of oral oestrogen and progesterone on liver function. You have discussed the next treatment plan with her. Please answer the following questions. A score of 0 points indicates that you complete disagree, and a score of 5 points indicates that you completely agree (the SMD-Q-Doc questionnaire).	
17	I have made it clear to the patient that taking oral oestrogen and progesterone is very important, and whether to continue treatment must be decided upon.
18	I would like to have a clear understanding of how the patient made the decision to terminate oestrogen and progesterone therapy.
19	I have informed the patient what different treatment options are available based on the current situation.
20	I accurately explained the pros and cons of different treatment options to the patient.
21	I helped the patient understand all the relevant information about the disease itself and oestrogen and progesterone treatment.
22	I asked the patient what treatment plan she was most willing to choose.
23	I discussed with the patient, and we comprehensively weighed the pros and cons of different treatment plans such as stopping the medication, adjusting the dosage, and using different formulations.
24	We made joint decisions about the next treatment plan.
25	I reached a consensus with the patient regarding whether she should receive oestrogen and progesterone treatment, as well as the methods and details of treatment.

1. Attitudes and the implementation propensity of the six steps of shared decision-making (SDM) in daily work [14]: (1) inviting patients to participate, (2) presenting all available options in simple and easy-to-understand language, (3) providing information on benefits and risks, (4) assisting patients in evaluating their options based on their goals/concerns, (5) facilitating deliberation and decision-making and (6) assisting in decision-making (Questions 10 to 15 in Table 1).
2. Potential barriers to the implementation of SDM: Participants were asked to rate a list of potential barriers summarized by previous systemic reviews of studies in English and French [15] and Chinese [16] (Question 16 in Table 1).
3. Effectiveness of SDM implementation strategies: All participants were asked to answer the nine questions in the Shared Decision-Making Questionnaire (SDM-Q-Doc) in the specific case of an endocrine disorder provided as a clinical scenario (for this study, we chose the scenario of a patient with Turner syndrome who needed oestrogen replacement therapy) (Questions 17 to 25 in Table 1). The SDM-Q-Doc is a 9-item measure of the decisional process in medical encounters from physicians’ perspectives. There are nine items in this single-dimensional self-assessment scale. Scoring is performed using a Likert-type scale, with 0 indicating ‘complete disagreement’, and 5 indicating ‘complete agreement’. The total score ranges from 0 to 45. A total score of 0 indicates the lowest perceived SDM level, and 45 indicates the maximum level. This scale has good acceptance, feasibility, and reliability [17]. The Chinese version of the SDM-Q-Doc is reliable and valid, and the internal consistency analysis yielded a Cronbach’s α of 0.867 and a test-retest reliability of 0.810 [18].
4. Demographic data included age, sex, education level, professional qualification, experience in the management of endocrine disorders and CME training (Questions 1 to 9 in Table 1).

### Eight-day online continuing medical education course

An eight-day online continuing medical education (CME) course was provided by faculty from Peking Union Medical College Hospital, a Class A tertiary comprehensive hospital committed to delivering state-of-the-art clinical care, conducting innovative scientific research, and providing rigorous medical education [19]. Endocrinologists who participate needed to pay a certain registration fee. The CME course covered significant fields of clinical endocrinology, including six sections for both common and rare diseases: (1) metabolic bone diseases; (2) disorders of abnormal glycolipid metabolism; (3) diseases of the hypothalamus and pituitary gland; (4) thyroid diseases; (5) adrenal diseases and hypertension; and (6) aberrant growth in children and adolescents. Each section included both keynote speeches and case-based learning. The primary focus of the keynote address was on the most recent advancements in epidemiology, pathophysiological mechanisms, clinical manifestations, techniques for diagnosis and therapeutic approaches to diseases. Case-based learning focused on one case at a time, reviewing the medical history and organizing group discussions on the issues that could arise during clinical diagnosis and treatment.

To evaluate the effects of the CME course on the effectiveness of SDM implementation, the SDM-Q-Doc was administered immediately before and after the training course regarding a specific case of an endocrine disorder, for which a clinical scenario was provided (for this study, we chose the scenario of a patient with multiple endocrine neoplasia type 1). In this part of the study, the inclusion criterion was endocrinologists who finished the full-term CEM course. The exclusion criteria were (1) early withdrawal from the CME course, (2) the inability to answer the questionnaires using a mobile device, and (3) no unique WeChat account registered. All participants were invited to complete the questionnaire within one week after the end of the CME course.

### Statistical analysis

Continuous data are presented as the mean ± standard deviation (SD) and were tested with the t test. Categorical data are presented as proportions and were tested with the chi-square test. The change in the SDM-Q-DOC (ΔSDM-Q-DOC) score was calculated as follows: the score at the end of the course minus the score at the beginning of the course. A paired-sample t test was used to compare the change in the SDM-Q-DOC score (ΔSDM-Q-DOC). Multiple stepwise linear regression was used to explore the influencing factors of ΔSDM-Q-DOC after the eight-day online CME course. All statistical computations were run using SPSS software version 22.0 for Windows (SPSS Inc., Chicago, IL, USA), and p < 0.05 was considered statistically significant.

## Results

### Demographic characteristics of the participants

A total of 512 specialists were invited to take part in the national survey, with 280 participants from 28 provinces of China responding to the self-administered questionnaire and completing every question (a response rate of 54.6%). Among the participants, 75.7% were female. The mean age was 38.0 ± 4.5 years. Furthermore, 62.5% of the participants had more than ten years of clinical experience. Other information on our participants’ characteristics is presented in Table 2. Of the physicians who participated in the national survey, 196 did not take the online CME course (non-CME group), and 84 registered for the online CME course (CME group).

**Table 2:** Characteristics of the participants in the national survey

Variable		Non-CME group No. (%) (n = 196)	CME group No. (%) (n = 84)	P value
Sex				
	Female	141 (71.9)	71 (84.5)	0.261
	Male	55 (28.1)	13 (15.5)	
Age group				
	20 ~ 29 yrs	4 (2.0)	0	0.306
	30 ~ 39 yrs	95 (48.5)	58 (69.0)	
	>= 40 yrs	97 (49.5)	26 (31.0)	
Education level				
	Bachelor’s degree	32 (16.3)	14 (16.7)	0.306
	Master’s degree	116 (59.2)	51 (60.7)	
	Doctoral degree	48 (24.5)	19 (22.6)	
Practice duration				
	< 5 yrs	22 (11.2)	6 (7.1)	0.306
	5 ~ 10 yrs	40 (20.4)	37 (44.0)	
	> 10 yrs	134 (68.4)	41 (48.8)	
Professional qualification				
	Professor	35 (17.9)	7 (8.3)	0.333
	Associate professor	92 (46.9)	27 (32.1)	
	Attending	57 (29.1)	48 (57.1)	
	Fellow	12 (6.1)	2 (2.4)	
Academic staff (Yes or No)		164 (83.7)	74 (88.1)	0.261
Annual number of rare disease cases				
	< 10	144 (73.5)	58 (69.0)	0.423
	10 ~ 50	49 (25.0)	25 (29.8)	
	50 ~ 100	3 (1.5)	1 (1.2)	
Annual number of MDT for rare endocrine diseases				
	< 10	160 (81.6)	61 (72.6)	0.261
	10 ~ 50	36 (18.4)	23 (27.4)	
Annual participation in CME for rare endocrine diseases				
	< 5	128 (65.3)	47 (56.0)	0.306
	5 ~ 10	56 (28.6)	28 (33.3)	
	> 10	12 (6.1)	9 (10.7)	
SDM-Q-DOC score, mean (SD) ^*^		84.4 (13.9)	87.3 (18.2)	0.147

* SDM-Q-DOC score =

Shared Decision-Making Questionnaire score

In the non-CME group, the mean age was 38.0 ± 4.5 years, and 71.9% (141/196) of the participants were female. A total of 59.2% of the participants had a master’s degree, and 24.5% had a doctoral degree. A total of 46.9% of the participants were associate professors, and 17.9% were professors. A total of 68.4% of the participants had a duration of practice of more than 10 years. A total of 83.7% pf the participants worked at academic medical centres. A total of 73.5% of the participants had fewer than 10 annual rare disease cases, and 81.6% had less than 10 annual multiple medical treatment (MDT) experiences. A total of 93.9% of the participants had participated in annual CME for rare endocrine diseases less than 10 times, and only 6.1% participated in annual CME for rare endocrine diseases more than 10 times.

Table 2 shows the demographics of the participants enrolled in the online CME course (CME group, n = 84). No characteristics were significantly different between the two groups.

## Endocrinologists’ perspectives of shared decision-making in daily practice

### Attitudes and the implementation propensity of SDM

Table 3 shows the participants’ knowledge scores and propensity of the six steps of SDM. There was an overall positive attitude towards SDM in clinical practice among the participants, with agreement ranging from 78.1 to 93.8%. There was moderate uncertainty regarding the six steps of implementation. The main uncertainties regarding the implementation steps included inviting patients to participate in SDM (16.3%), assisting in decision-making (15.3%), facilitating deliberation and decision-making (13.7%), and providing information on benefits and risks (12.6%).

**Table 3:** The implementation tendency of the six steps of shared decision-making (SDM) in daily practice in the national survey group (n = 280)

1.	Propensity to invite patients to participate in SDM	No. (%)
	Yes	218 (77.8)
	No	16 (5.7)
	uncertain	46 (16.4)
2.	Present all available options in simple, easy-to-understand language	
	Yes	262 (93.6)
	No	2 (0.7)
	uncertain	16 (5.7)
3.	Provide information on benefits and risks	
	Yes	237 (84.6)
	No	7 (2.5)
	uncertain	36 (12.9)
4.	Assist patients in evaluating options based on their goals/concerns	
	Yes	260 (92.9)
	No	6 (2.1)
	uncertain	14 (5.0)
5.	Facilitate deliberation and decision-making	
	Yes	235 (83.9)
	No	6 (2.1)
	uncertain	39 (13.9)
6.	Assist with decision-making	
	Yes	230 (82.1)
	No	7 (2.5)
	uncertain	43 (15.3)

### Potential barriers to implementing SDM in clinical practice

In Table 4, providers’ views of potential barriers to implementing SDM in the clinical practice of endocrinology are listed. The highest agreement was shown for the following perceived barriers in implementing SDM in clinical practice: that SDM would be time-consuming (63.8%), that there would be unequal information between doctors and patients (62.8%) and that there would be a lack of technical support and training. Most participants did not doubt the scientific basis of SDM (95.9%) or the contribution of SDM to improving clinical outcomes (91.8%). Participants agreed that they were competent regarding their communication skills (91.8%) but were worried about patients’ willingness to be involved in SDM (83.1%) or trust in the effect of SDM on improving treatment outcomes (83.2%). A lack of medical knowledge of patients (77.6%) was also an important potential barrier in the implementation of SDM.

**Table 4:** Providers’ views of practical barriers to SDM in the management of rare endocrine disorders in the national survey group (n = 280)

Questions about potential barriers to SDM implementation in daily practice	No. (%)	
	Yes	No
Q1: Too occupied to have enough time for SDM in daily work	178 (63.6)	102 (36.4)
Q2: Lack of technical support and training for SDM	151 (53.9)	129 (46.1)
Q3: Doubt about the scientific basis of SDM	12 (4.3)	268 (95.7)
Q4: Doubt about the effects of SDM on improving treatment outcomes	23 (8.2)	257 (91.8)
Q5: Lack of competency and communication skills for SDM	23 (8.2)	257 (91.8)
Q6: Patients unwilling to participate in SDM	61 (21.8)	219 (78.2)
Q7: Patients do not believe that SDM could improve their treatment outcomes	47 (16.8)	233 (83.2)
Q8: The lack of medical knowledge of patients makes it difficult for them to participate in SDM	63 (22.5)	217 (77.5)
Q9: Unequal information between doctors and patients	175 (62.5)	105 (37.5)

### Effectiveness of SDM implementation strategies

In the scenario of a specific case of an endocrine disorder, the SDM-Q-DOC score was 84.4 ± 13.9 in the non-CME group and 87.3 ± 18.2 in the CME group (p = 0.147).

### Online CME has positive effects on physicians’ perspectives during the process of SDM

In the CME group (n = 84), the SDM-Q-Doc score increased from 87.3 ± 18.2 at baseline to 93.0 ± 9.3 at the end of the 8-day online CME training (p = 0.003, paired t test). Multiple stepwise linear regression analysis revealed that the participants’ age, sex, education level, practice duration, annual number of patients with rare diseases, annual number of patients requiring MDT or CME were not significantly related to the ΔSDM-Q-DOC after the eight-day online CME course (all p > 0.05).

## Discussion

In this pilot study, we aimed to evaluate the perspectives of shared decision-making among Chinese endocrinologists and the effects of an online CME course. Our results found that (1) Chinese endocrinologists had a generally positive attitude towards SDM. (2) There were also several uncertainties regarding the implementation steps of SDM. The main perceived barriers included time constraints, information inequality between doctors and patients, and a lack of technical support and training for SDM. (3) Regardless of a physician’s educational background or prior professional experience, CME may help to improve their perspectives regarding SDM.

The landscape of clinical endocrinology is constantly evolving. On the one hand, biomedical research advances, including genomics, have enabled more hereditary endocrine diseases to be recognized. On the other hand, updated information from clinical trials has provided various evidence for managing endocrine diseases. In their daily work, it has become challenging for endocrinologists to share new evidence, evaluate the advantages and disadvantages of new regimens in combination with patients’ preferences, and make shared decisions with patients [6]. In the field of diabetes, there are various large-scale research data, and the development of new medications continues to evolve constantly. Sufficient information and data are available for patients with diabetes and their doctors to become proficient at jointly making decisions. However, many constraints remain on how SDM is utilized in clinical practice [20], similar to the situation encountered in the clinical diagnosis and treatment of papillary thyroid cancer and patients treated with growth hormone [21, 22]. Recognizing that with the best current evidence, there is no clear best choice for a particular regimen is the first step in preparing to make a share decision. In our national survey, Chinese endocrinologists had a generally positive attitude towards SDM in clinical practice. There were also several uncertainties regarding the implementation steps of SDM. The uncertainties come from both limitations in the state-of-the-art evidence and the need for more patient preferences. To overcome these uncertainties, doctors need training in communication skills and providing patients with the necessary information, education, and tools to enable them to participate meaningfully in decision-making processes. Understanding patients’ values, beliefs and preferences will further help identify the most appropriate treatment options that align with their personal values.

Continued medical education plays a crucial role in enhancing physicians’ professional knowledge and shared decision-making skills. Several recent studies have focused on CME interventions aiming to promote implementation skills in decision-making among physicians [23], utilizing randomized controlled trials and before-and-after designs [24–27]. These interventions primarily involved face-to-face training, specialized lectures, workshops incorporating video modelling of ideal behaviour, and role-play exercises. The CME courses varied from a few hours to six months. Encouragingly, these interventions resulted in improvements in physicians’ communication behaviour and clinically relevant enhancements in patient orientation [26, 28]. Contrary to previous studies that focused mainly on improving communication behaviour and patient orientation, our study specifically evaluated the effects of an eight-day online CME program on the SDM-Q-DOC scores of specialists with years of practice. We found no significant associations between participants’ demographic characteristics (age, sex, education level), practice duration, or engagement in other professional activities (such as rare endocrine disease management or participation in multidisciplinary team meetings) and changes in their SDM-Q-DOC scores.

The present study has several limitations that warrant consideration. First, a potential limitation is the lack of participant representativeness among all endocrinologists, which may introduce responder bias. Additionally, as our data were obtained through self-administered questionnaires, it is important to acknowledge the possibility of information bias. Another limitation lies in the use of the SMD-Q-Doc score as the main outcome measure, which was assessed within a simulated clinical scenario rather than a real clinical setting during the prospective study. Furthermore, the absence of specific questions pertaining to participants’ prior shared decision-making (SDM) training in the questionnaire introduces a potential confounding bias. Finally, it is important to note that this study did not include a questionnaire-based evaluation of SDM among patients. Therefore, further evaluations of SDM between doctors and patients are necessary to identify obstacles to SDM in clinical practice and develop corresponding improvement strategies.

In conclusion, Chinese endocrinologists had a generally positive attitude towards SDM in clinical practice. Regardless of a physician’s educational background or prior professional experience, CME may help to improve their perspective regarding SDM.

## Data Availability

The datasets used and/or analysed during the current study are available from the corresponding author upon reasonable request.
